# Autonomic dysreflexia: the concealed killer behind recurrent cerebral hemorrhage in spinal cord injury—a case report with management insights

**DOI:** 10.3389/fnins.2026.1800186

**Published:** 2026-05-15

**Authors:** Nengzhang Tang, Bi'e Zheng, Jun Ni, Yimiao Xie, Lifang Zhang, Qiong Han, Limin Sun

**Affiliations:** 1 Department of Rehabilitation, National Regional Medical Center, Binhai Campus of the First Affiliated Hospital, Fujian Medical University, Fuzhou, China; 2 Department of Rehabilitation, the First Affiliated Hospital, Fujian Medical University, Fuzhou, China

**Keywords:** autonomic dysreflexia, blood pressure variability, case report, intracerebral hemorrhage, spinal cord injury

## Abstract

Autonomic dysreflexia (AD) is a potentially life-threatening complication of high-level spinal cord injury (SCI), marked by paroxysmal hypertension. Although cerebrovascular events can be triggered by severe hypertension, the direct association between AD and intracerebral hemorrhage (ICH) necessitates increased clinical awareness. We present a case of a 71-year-old male with a complete C3 SCI (American Spinal Injury Association Impairment Scale grade A). On May 24, 2025, the patient developed an acute episode of AD following defecation, characterized by a sudden, severe headache and transient loss of consciousness, with elevated blood pressure (BP) of 178/101 mmHg. Emergency computed tomography revealed a right occipital ICH (3.6 × 1.8 cm) with concomitant subarachnoid hemorrhage. A follow-up cranial imaging examination on June 21, 2025, revealed a new contralateral hematoma (3.4 × 3.0 cm) in the left frontal lobe. Notably, a follow-up 24-h ambulatory blood pressure monitoring performed between the two hemorrhagic events (on June 20, 2025) revealed markedly elevated blood pressure variability, with a systolic BP standard deviation of 32.7 mmHg (compared with 31.8 mmHg recorded before the initial hemorrhage). Pre-event 24-h ambulatory blood pressure monitoring performed on March 21, 2025, had already demonstrated marked blood pressure variability (BPV), which may reflect the patient’s underlying autonomic dysregulation. The association between this extreme BPV and the subsequent ICH remains a subject for hypothesis generation. The hematomas resolved following a regimen of antihypertensive therapy (nitrendipine), osmotic diuresis (mannitol), and meticulous management of triggering factors. This case demonstrates that AD in high cervical SCI can precipitate severe ICH, with extreme BPV potentially serving as a synergistic risk factor. Clinicians should maintain high vigilance for new-onset severe headache in patients with SCI at or above T6, ensuring prompt BP assessment and identification of AD triggers. Future studies are required to investigate whether long-term BPV stabilization could mitigate hemorrhagic risk in this population.

## Introduction

Autonomic dysreflexia (AD) is a potentially fatal complication of spinal cord injury (SCI), affecting over two-thirds of patients with an injury at or above the sixth thoracic vertebral level (≥ T6). It is marked by acute hypertensive episodes triggered by noxious or non-noxious stimuli below the neurological level (Ebrahimi et al., 2023; Pavese and Kessler, 2023; Calderón-Juárez et al., 2024; Walter et al., 2025). Marked hypertension associated with AD significantly elevates cerebrovascular accident risk (Cowan et al., 2020; Bilgin Badur et al., 2025). Blood pressure variability (BPV), characterized by temporal fluctuations in BP over time, serves as an independent predictor of poor outcomes in traumatic brain injury (Svedung Wettervik et al., 2020) and spontaneous intracerebral hemorrhage (ICH) (Jafari and Damani, 2020); however, it remains understudied in AD-related cerebrovascular events post-SCI.

We report a case of a patient with a complete C3 SCI who experienced recurrent ICH triggered by AD during defecation. We hypothesize that extreme BPV may indicate severe autonomic instability, and its role in directly increasing cerebrovascular vulnerability in this population requires further clarification and investigation.

Previous reports have confirmed the association between AD and severe cerebrovascular events, including ICH and seizures (Dolinak and Balraj, 2007; Hartman et al., 2021). However, the precise function of extreme BPV in this context remains underexplored. The primary contribution of this case report is to establish AD as a cause of ICH, which is already recognized, and to present a hypothesis-generating observation concerning the potential role of extreme BPV as a marker of vulnerability in a patient who suffered recurrent ICH. This study contributes to the literature by providing quantitative BPV indices before an AD-triggered ICH event, a detail largely overlooked in previous case descriptions.

## Case report

**Figure 1:**
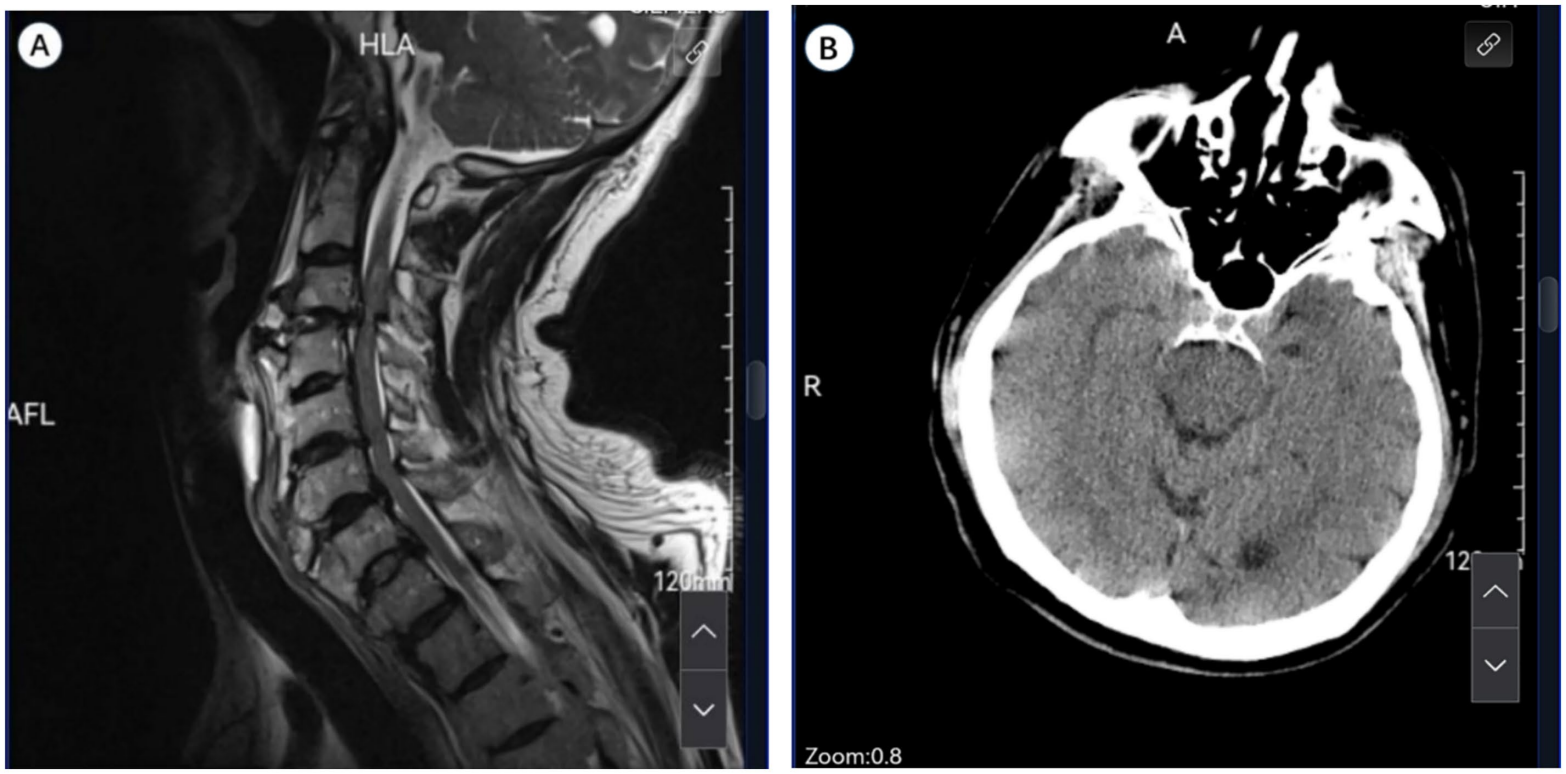
**(A)** Sagittal T2-weighted cervical spine MRI post-injury demonstrates spinal cord injury at C3-C4. **(B)** Initial emergency cranial CT reveals no intracranial abnormalities.

We report a case of a previously independent 71-year-old male with a history of hypertension who was admitted on December 10, 2024, after experiencing a mechanical fall. Magnetic resonance imaging (MRI) revealed an SCI at the C3**–**C4 level (Figure 1), and posterior cervical laminoplasty with lateral mass screw fixation was performed. Preliminary cranial computed tomography (CT) revealed no sign of intracranial pathology. The patient had no previous history of coagulopathy or cerebrovascular events. He was not receiving any antiplatelet or anticoagulant therapy.

*Informed consent*: Written informed consent was obtained from the patient for publication of this report and the accompanying images.

### Rehabilitation and admission status

On February 27, 2025, the patient commenced inpatient rehabilitation. Examination confirmed a complete C3 SCI (ASIA Impairment Scale grade A with an indwelling urinary catheter *in situ*. A structured bowel and bladder program was initiated to prevent AD triggers.

### Autonomic function assessment and preliminary events

**Figure 2:**
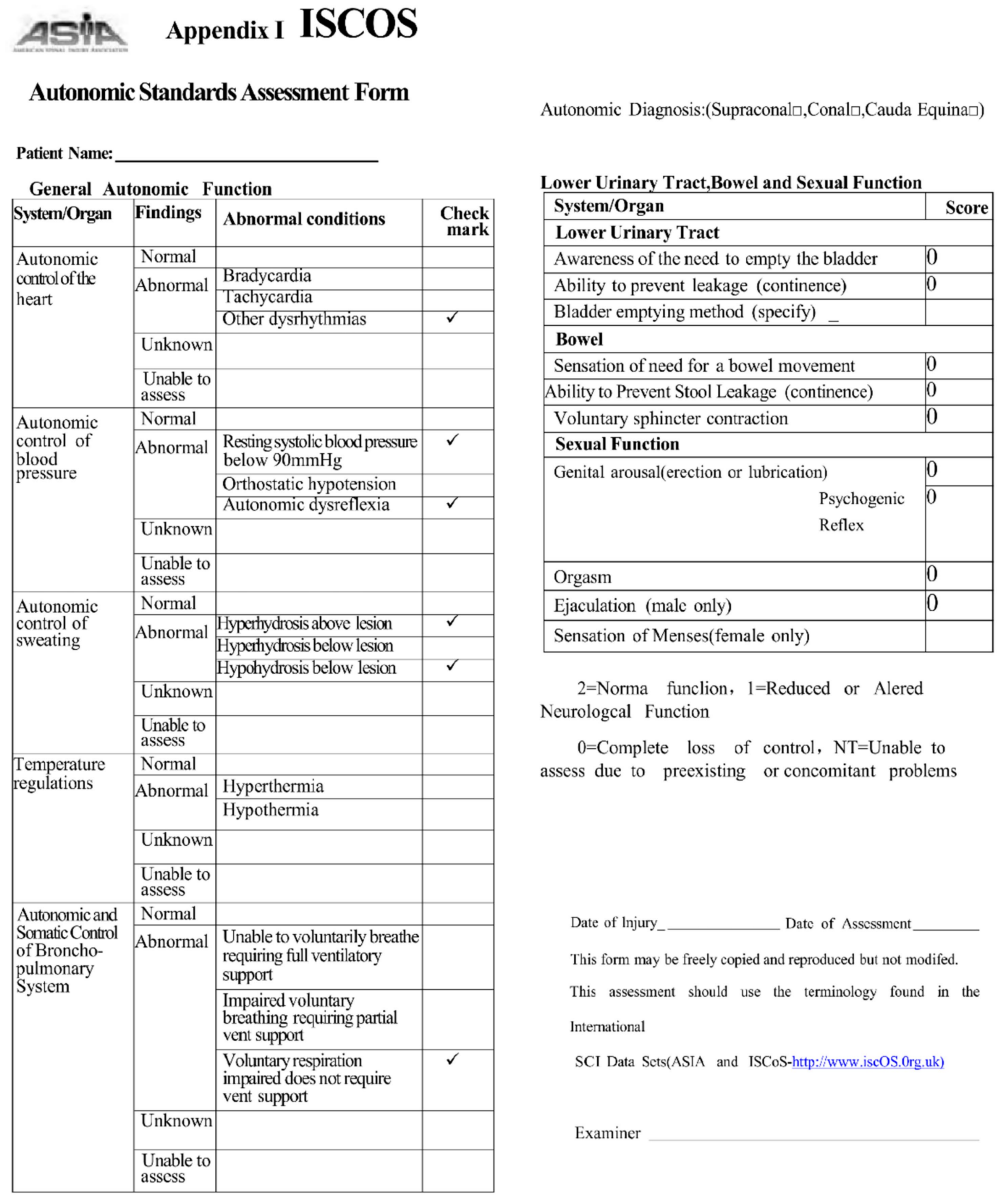
International standards to document remaining autonomic function after spinal cord injury (ISAFSCI) assessment form, Appendix I.

**Figure 3:**
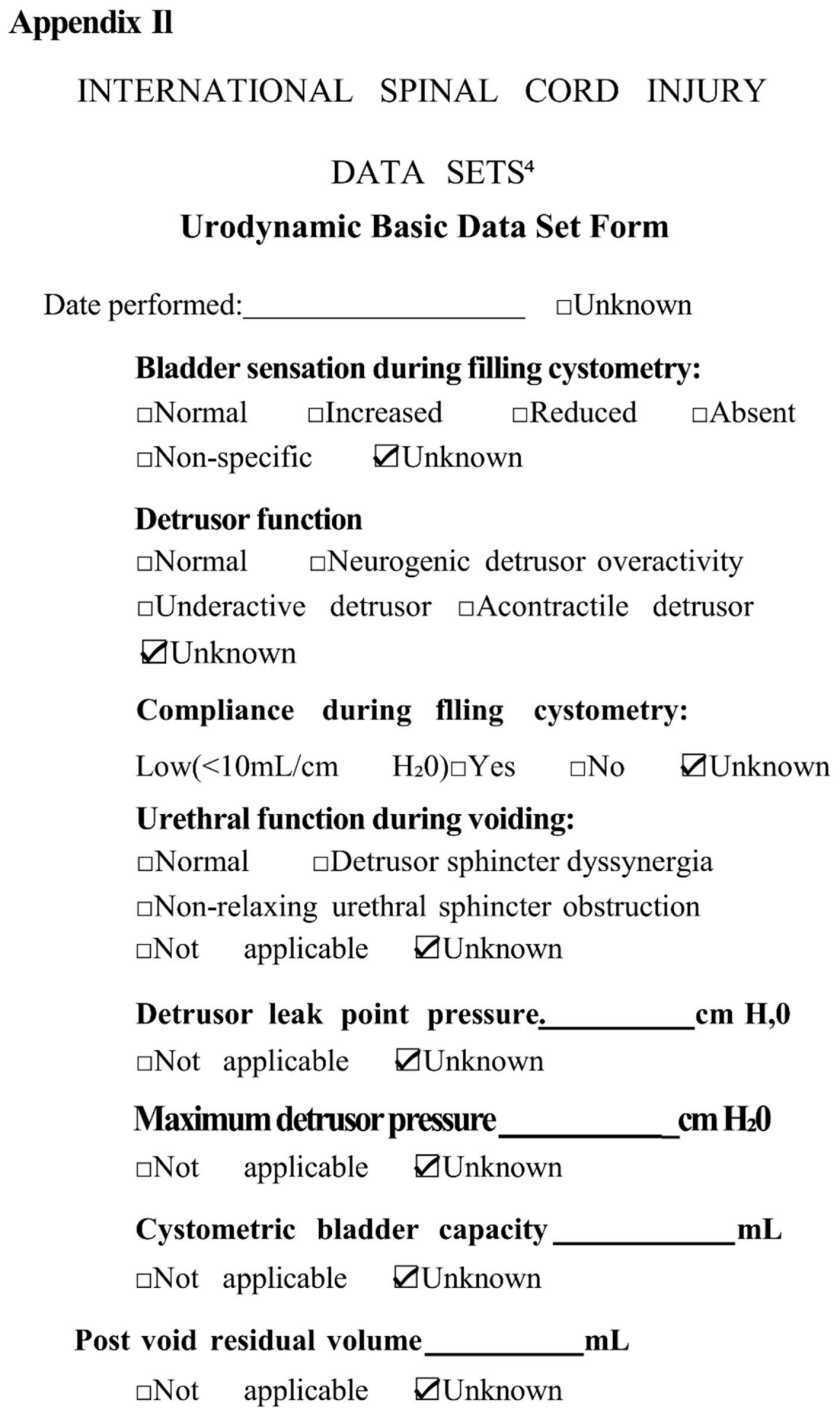
ISAFSCI assessment form Appendix II.

The formal assessment of autonomic function, using the International Standards to document remaining Autonomic Function after Spinal Cord Injury (ISAFSCI) (Wecht et al., 2021), validated multi-system autonomic dysfunction (Figures 2, 3).

**Figure 4:**
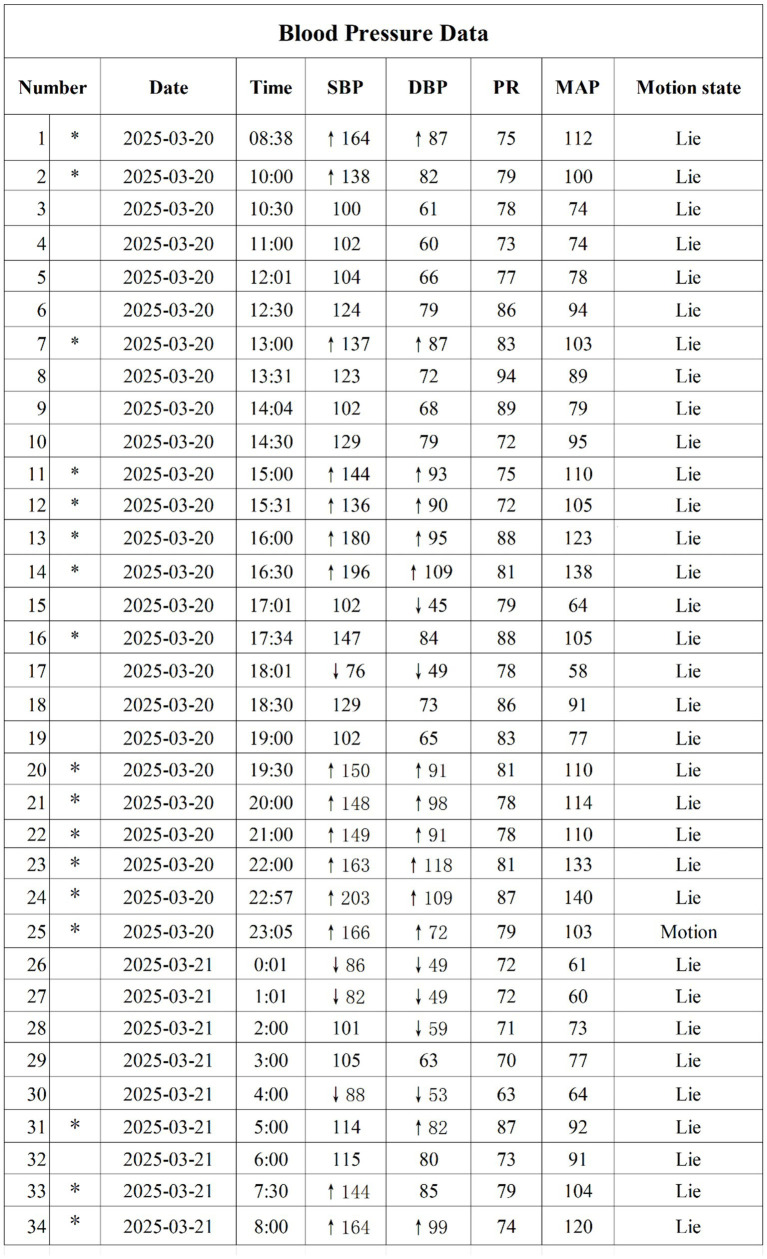
24-h ambulatory blood pressure monitoring (March 21, 2025). SBP, systolic blood pressure; DBP, diastolic blood pressure; PR, pulse rate; MAP, mean arterial pressure; SD, standard deviation; CV, coefficient of variation.

Pre-event 24-h ambulatory blood pressure monitoring (ABPM) was performed during a stable period without acute AD episodes or ICH. It revealed marked BPV: systolic BP (SBP) ranged from 76 to 203 mmHg, and diastolic BP (DBP) ranged from 45 to 119 mmHg; calculated indices included SBP standard deviation (SD) = 31.8 mmHg and SBP coefficient of variation (CV) = 24.5% (Figure 4).

### Acute AD episode and ICH diagnosis

**Figure 5:**
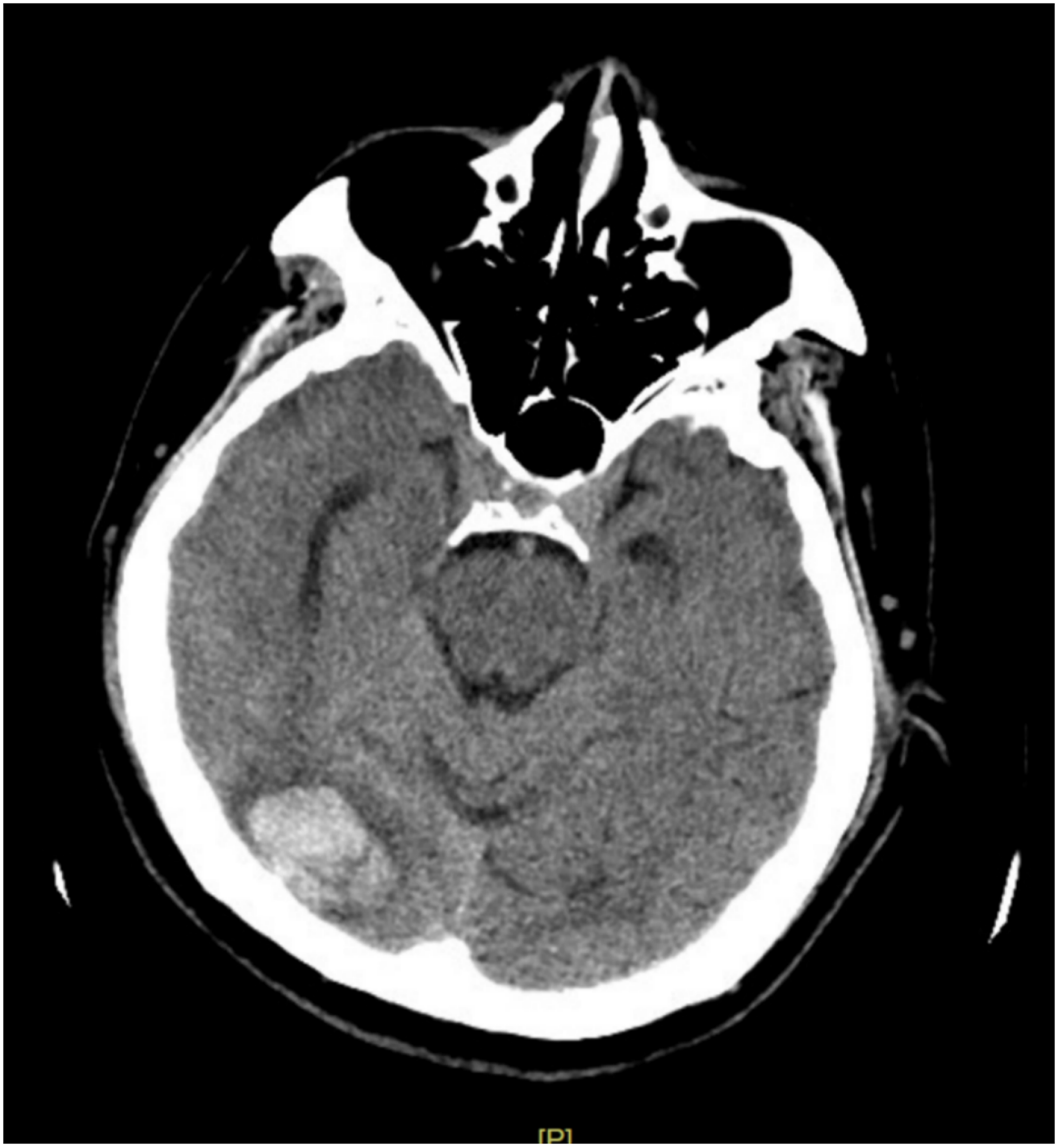
Emergency cranial CT scan (May 24, 2025) reveals a hemorrhage in the right occipital lobe.

On May 24, 2025, the patient developed an acute episode of AD immediately after defecation, accompanied by an acute severe headache and transient loss of consciousness. BP was 178/101 mmHg. Oral nitrendipine (10 mg) was administered for acute BP control due to the unavailability of guideline-preferred, short-acting agents. Emergency cranial CT revealed a right occipital ICH (Figure 5).

### Management and clinical course

Following neurosurgical consultation, a conservative management strategy was implemented:

- *BP management:* The target SBP was set below 140 mmHg, sustained with oral nitrendipine as required.
- *Intracranial pressure management:* 20% mannitol was administered to mitigate cerebral edema.
- *AD trigger management:* Potential triggers were eliminated. Urinary catheter patency was ensured. A structured, preventive bowel regimen was instituted, comprising a high-fiber diet, adequate fluid intake, and a biweekly scheduled bowel evacuation regimen. To reduce rectal stimulation and AD risk, the program employed gentle abdominal massage combined with a pre-lubricated glycerin (20 mL) administered only after verifying the absence of digital impaction and with continuous BP surveillance. Oral lactulose (15 mL twice daily) was added to maintain stool softness and prevent straining.
- *Cerebrovascular spasm prophylaxis:* nimodipine was commenced.

**Figure 6:**
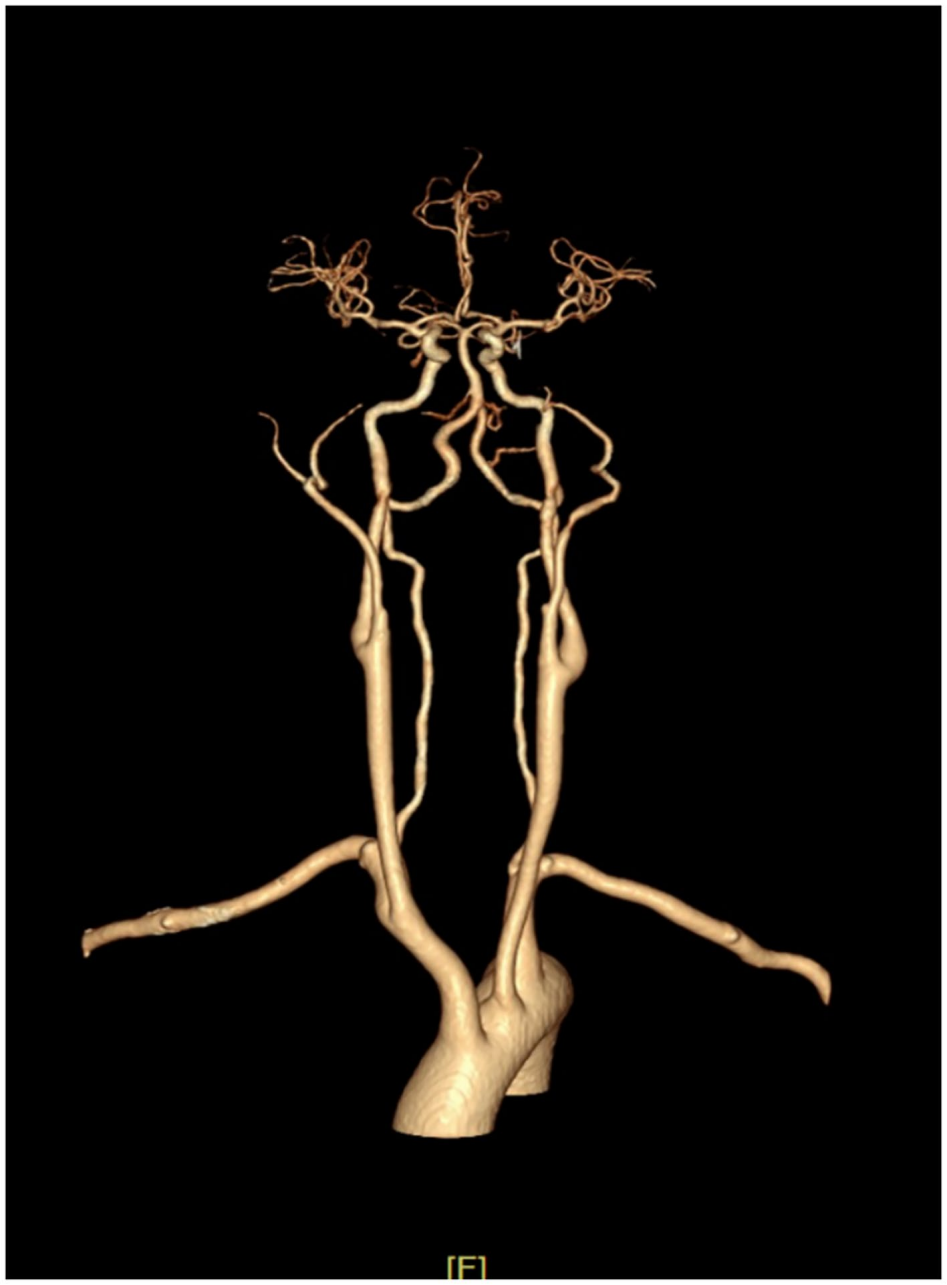
Head and neck CTA (May 27, 2025) depicting a congenitally smaller V4 segment of the left vertebral artery compared to the contralateral side with mild arteriosclerosis.

Cranial and cervical computed tomography angiography (CTA) revealed a congenitally slender left vertebral artery V4 segment and mild atherosclerosis (Figure 6).

**Figure 7:**
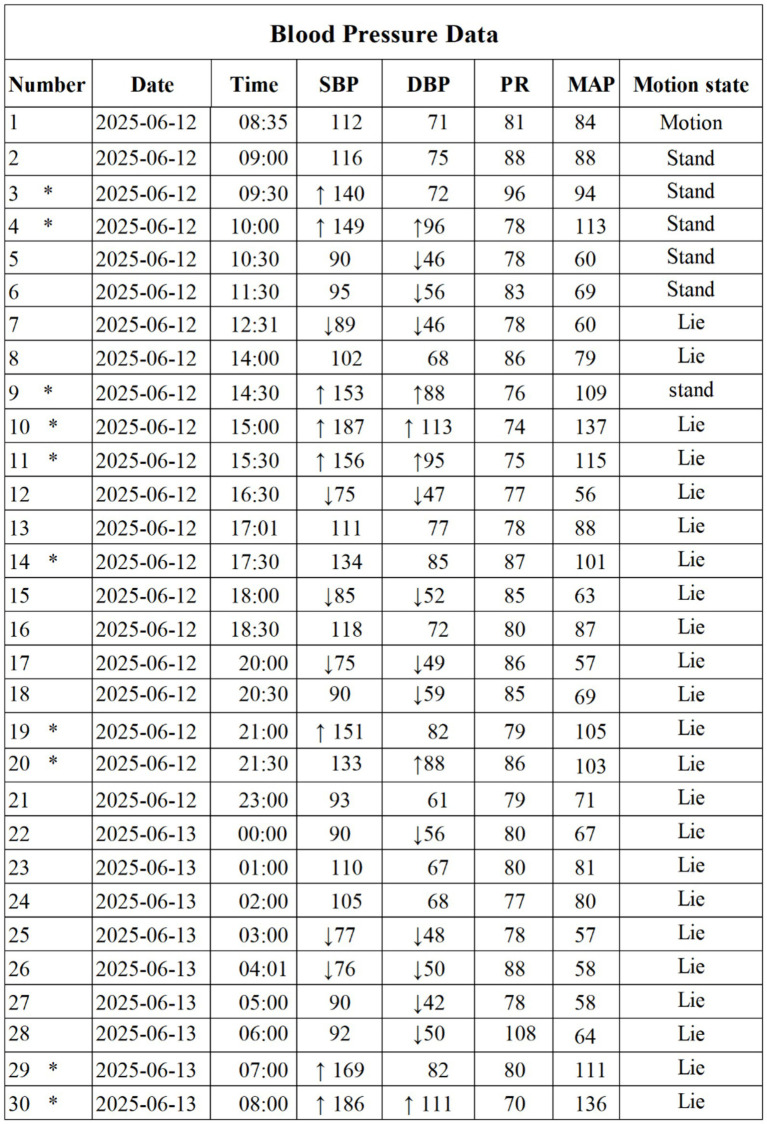
24-h ambulatory blood pressure monitoring (June 20, 2025). SBP, systolic blood pressure; DBP, diastolic blood pressure; PR, pulse rate; MAP, mean arterial pressure; SD, standard deviation; CV, coefficient of variation.

On June 20, 2025, follow-up 24-h ABPM indicated marked BP fluctuations (Figure 7), with a systolic blood pressure peak of 187 mmHg, a trough of 75 mmHg, and calculated indices of SBP SD = 32.7 mmHg and SBP CV = 28.4%. Notably, this ABPM recording (obtained on June 20, 2025, 1 day before MRI detection of the new left frontal hematoma on June 21, 2025) demonstrates extreme BPV (SBP SD 32.7 mmHg). This close temporal proximity highlights the persistent and unstable hemodynamic conditions that may lead to recurrent cerebrovascular events in this high-risk population.

**Figure 8:**
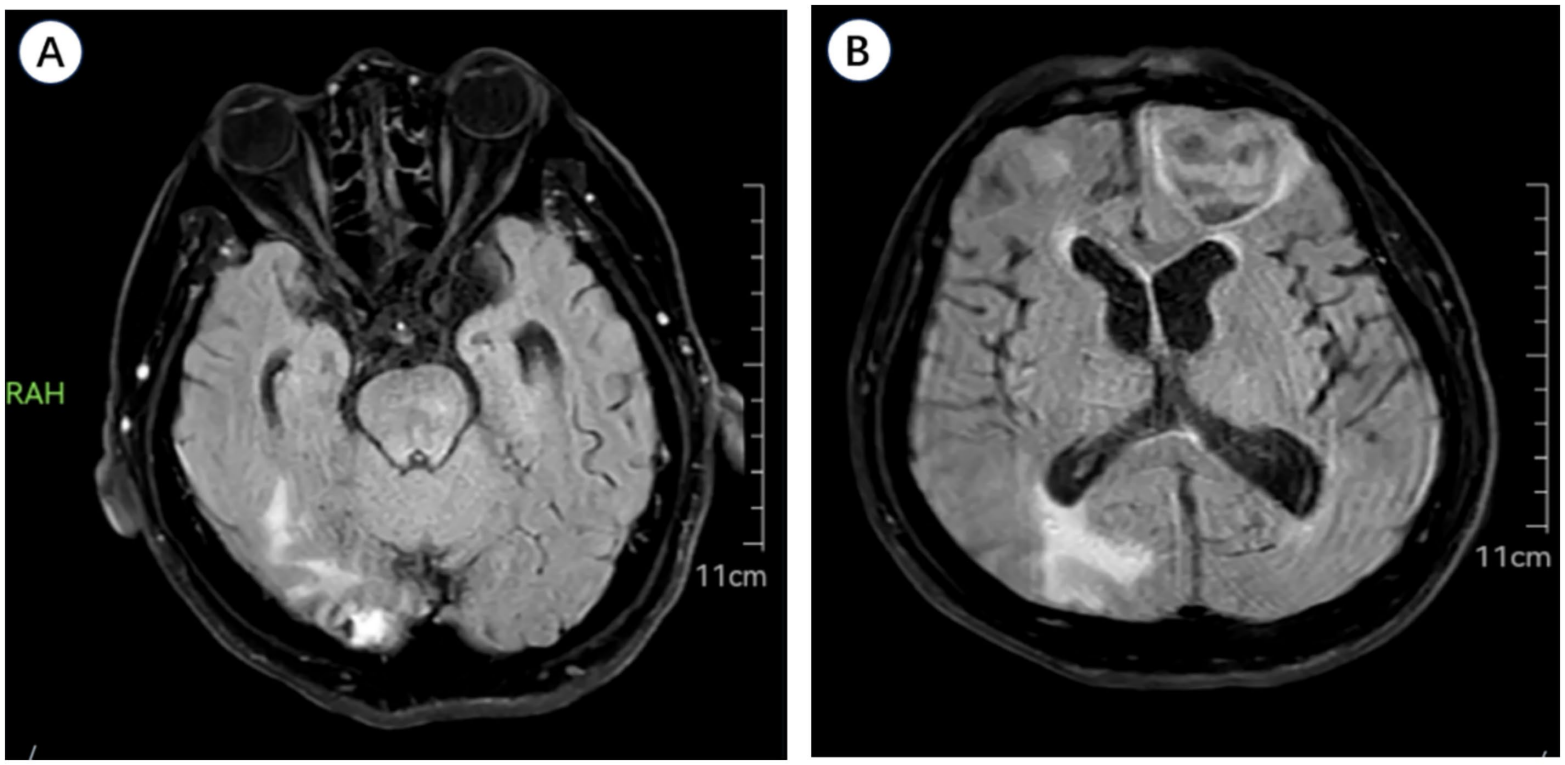
Cranial MRI (June 21, 2025) depicting **(A)** partial absorption of the right occipital lobe hematoma and **(B)** a new hematoma in the left frontal lobe.

During a follow-up cranial imaging examination on June 21, 2025, a new hematoma (3.4 × 3.0 cm) was identified in the left frontal lobe (Figure 8).

**Figure 9:**
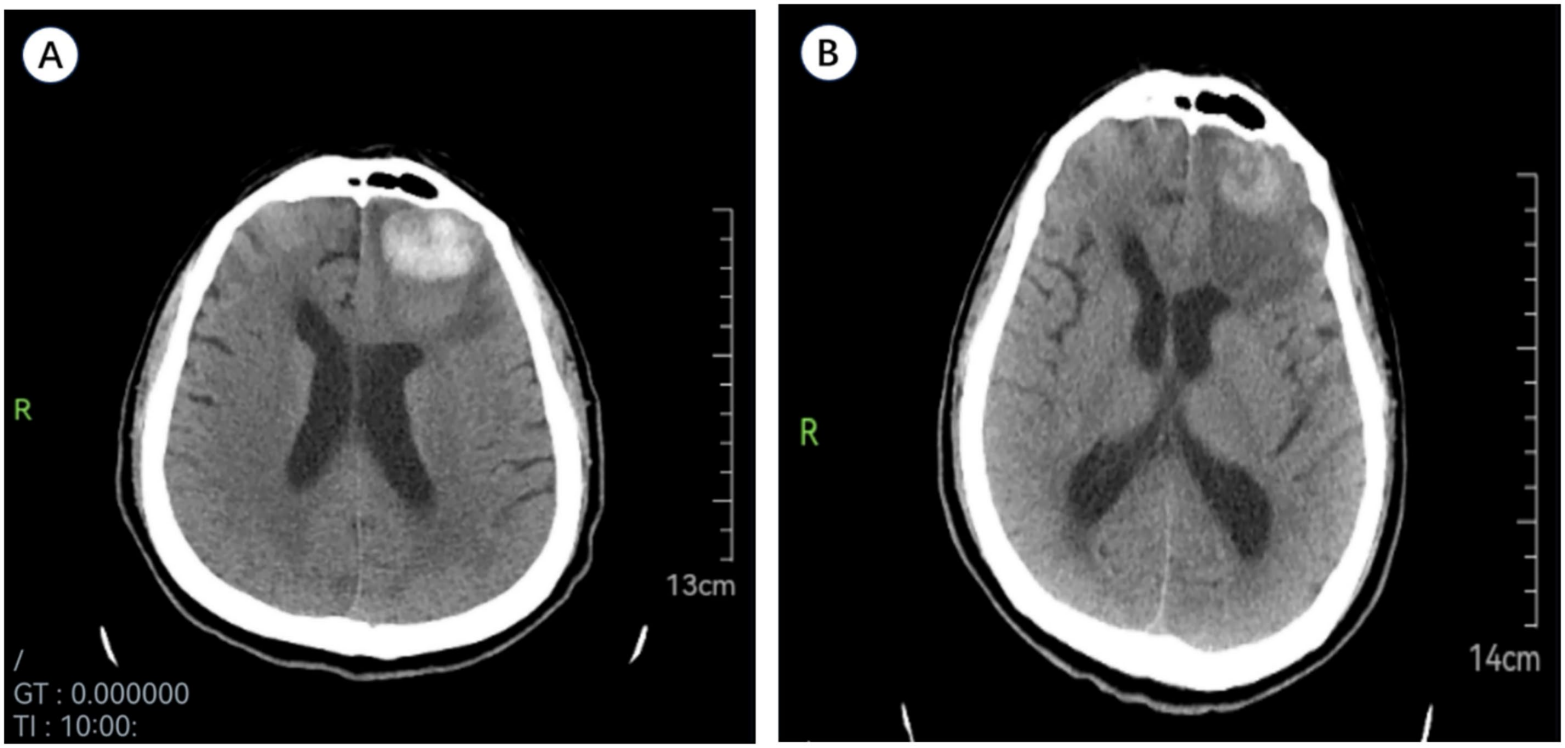
Follow-up cranial CT scans on **(A)** June 30, 2025, and **(B)** July 8, 2025, depicting a progressive reduction in the size of the left frontal lobe hematoma.

Conservative management with serial neuroimaging surveillance was recommended (Figure 9).

### Diagnostic assessment

*Diagnostic testing:* Cranial and cervical CT angiography ruled out aneurysm, arteriovenous malformation, or large vessel stenosis.

*Diagnostic challenges:* Cerebral amyloid angiopathy (CAA) was considered due to the patient’s age (71 years) and lobar hemorrhage locations. MRI examination was initially attempted, and essential axial T2/FLAIR sequences were successfully acquired (Figure 8). However, further characterization using susceptibility-weighted imaging (SWI) was discontinued due to the patient’s inability to endure prolonged scanning in supine positioning secondary to severe orthostatic hypotension and pain, precluding a safe and motionless examination. Given his recorded severe autonomic instability, symptoms of orthostatic intolerance markedly deteriorated after the initial 4–5 min of scanning. Prolonging the examination was therefore deemed clinically unsafe due to the risk of precipitating syncope or severe hypertensive rebound. This remains a significant diagnostic limitation.

*Diagnosis (including other diagnoses considered)*

- Primary diagnosis: Recurrent ICH secondary to AD triggered by bowel distension.
- Differential diagnoses considered: (1) Hypertensive ICH (unlikely due to mild atherosclerosis on CTA and lack of deep hemorrhages); (2) cerebral amyloid angiopathy (possible, but unverified without SWI); (3) structural lesion (excluded by CTA).

*Therapeutic intervention:* Management complied with the Consortium for Spinal Cord Medicine guidelines for acute AD (Krassioukov et al., 2021).

*Non-pharmacologic:* Immediate upright positioning, loosening of restrictive clothing/devices, and identification/removal of triggers.

*Pharmacologic (acute):* In the absence of short-acting agents such as sublingual nifedipine or nitroglycerin spray, oral nitrendipine 10 mg was administered as a one-time dose.

*Ongoing management:* A prophylactic bowel regimen was instituted. A 20% mannitol solution was administered. Nimodipine was administered for vasospasm prophylaxis.

*Changes in therapeutic intervention (with rationale):* No changes were made to the acute AD treatment protocol. The preventive bowel regimen was commenced after the first ICH and continued. No antihypertensive maintenance therapy was prescribed post-discharge due to recorded baseline supine hypotension during rehabilitation, with morning SBP readings often between 90 and 100 mmHg. Commencing daily antihypertensive therapy would have presented an intolerable risk of exacerbating orthostatic intolerance and precipitating falls or cerebral hypoperfusion during upright mobilization.

### Outcomes and follow-up

*Clinician-assessed outcomes:* No focal neurologic deficits were observed. Serial imaging demonstrated gradual hematoma resorption.

*Important follow-up diagnostic results:* On June 21, 2025, a follow-up MRI unexpectedly revealed a new left frontal ICH, which was clinically asymptomatic (not associated with new-onset headache, focal neurological deficits, or altered consciousness).

*Intervention adherence and tolerability:* The patient complied completely with the prescribed bowel regimen. He exhibited good tolerance to a single dose of nitrendipine. Adherence was evaluated through nursing records and direct patient interviews.

*Adverse and unanticipated events:* The newly identified left frontal ICH was an unexpected asymptomatic event. No adverse events attributable to pharmacologic interventions were observed.

**Figure 10:**
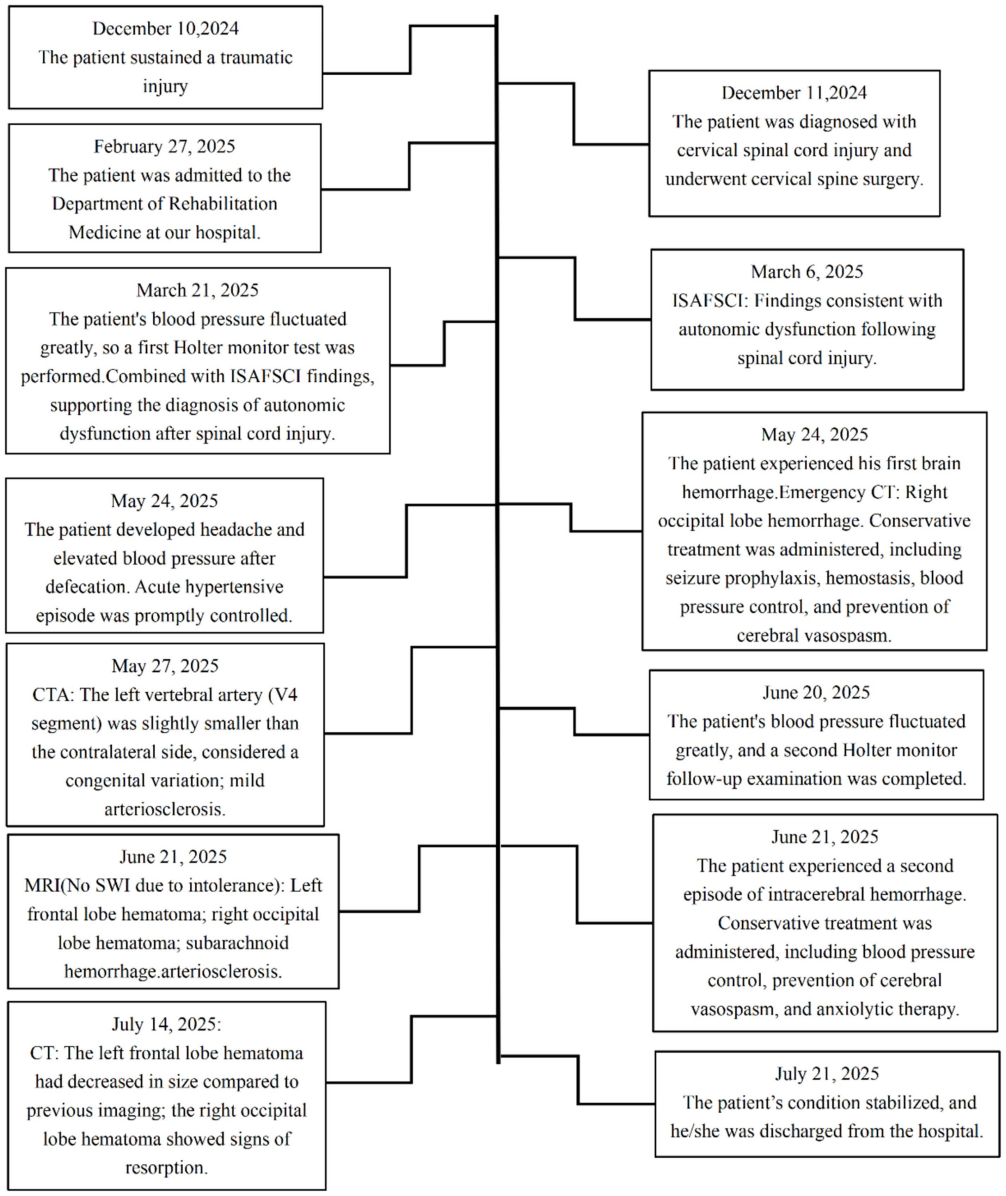
Patient timeline. CT, computed tomography; CTA, computed tomography angiography; MRI, Magnetic resonance imaging; ISAFSCI, International Standards to document remaining Autonomic Function after Spinal Cord Injury; ASIA, American Spinal Injury Association Impairment Scale; ABPM, ambulatory blood pressure monitoring; AD, autonomic dysreflexia; BPV, blood pressure variability.

Figure 10 summarizes the course of the disease.

## Discussion

Although the clinical manifestations of autonomic dysreflexia may differ, patients typically exhibit a sudden, severe headache associated with significant hypertension. Any patient with paraplegia or quadriplegia presenting with complaints of severe headache or found unconscious should promptly be screened for potential autonomic dysreflexia by measuring their blood pressure. AD following SCI is a potentially fatal complication. Hartman (Hartman et al., 2021) reported a case of AD in a young man with complete SCI, leading to epileptic seizures. Dolinak and Balraj (2007) reported a case of a 62-year-old man with quadriplegia following a diving accident. He developed AD and severe hypertension during his hospitalization. His nervous system deteriorated, and he died thereafter. The antemortem imaging examination revealed a large amount of hypertensive ICH in the right caudate nucleus. Pollifrone et al. (2021) examined a patient with post-SCI quadriplegia and Takotsubo cardiomyopathy following a severe episode of AD. Trønnes and Berg (2012) reported that a boy with a high SCI developed severe bradycardia resulting from AD induced by bladder distension, culminating in asystole and pulseless electrical activity.

### Complexity and recurrence

AD results from dysregulation of the autonomic system, causing an uncoordinated response to noxious stimuli below the level of a spinal cord injury, potentially presenting as BP instability, including hypotension, orthostatic hypotension, and abnormal autonomic reflexes, which may coexist. This patient experienced concurrent hypotensive and hypertensive events. BPV was notably significant. The patient’s pre-existing chronic hypertension probably contributed to baseline vascular fragility. Although AD served as the acute trigger, the underlying hypertensive vasculopathy may have lowered the threshold for ICH occurrence. The interaction between chronic hypertension, extreme BPV, and episodic AD-induced pressure surges establishes a notably high-risk condition that necessitates additional research. Paroxysmal hypertension, orthostatic hypotension, and supine hypotension coexisted and alternated. The cardiovascular and cerebrovascular risks significantly exceed those associated with uncomplicated hypertension (the correlation between BP fluctuations and arteriosclerosis and accelerated degeneration of elastic connective tissue due to aging, increasing the risk of cerebral hemorrhage), complicating clinical management.

This case was managed according to the established protocols for managing AD. Management adhered to established guidelines (Krassioukov et al., 2021) with non-pharmacologic interventions and nitrendipine for acute BP control due to the unavailability of short-acting agents.

AD may recur within 1–2 weeks following SCI, accompanied by AD and cerebral hemorrhage. Close observation, monitoring of BP and BPV, and follow-up head CT are essential (it may be an asymptomatic occult cerebral hemorrhage, as in this case). Additionally, susceptibility-weighted imaging (cerebral amyloidosis) should be enhanced if required, and magnetic resonance angiography (aneurysm, arteriovenous malformation, and cavernous hemangioma), magnetic resonance venography (sinus venous thrombosis), and other related examinations should be performed to exclude other cerebrovascular diseases. The coagulation function must be assessed and withhold anticoagulation therapy. Problems must be identified promptly and addressed immediately to avoid worsening the condition and causing fatal harm to the patient. The case reported in this study was promptly identified and treated. Neurology and neurosurgery teams were consulted, and conservative treatment was recommended. There was no evident worsening of neurological deficits.

### Temporal association between AD and ICH in the present case

Cervical SCI disrupts descending inhibitory pathways to the sympathetic preganglionic neurons. Stimulations of visceral afferents (from a distended bowel) may initiate an unregulated sympathetic storm, potentially surpassing cerebral autoregulatory mechanisms and precipitating vascular injury (Wulf and Tom, 2023; Trueblood et al., 2024). The temporal correlation between the trigger (defecation), the sudden increase in BP, and the neurological symptoms in this instance strongly substantiates AD as the initiating factor. Although occipital lobe hemorrhage is an uncommon site of hypertensive hemorrhage, it aligns with the mechanism of systemic BP elevation caused by AD. The reported mean SBP during AD-associated stroke is 187 mmHg (Wan and Krassioukov, 2014), whereas it was 178 mmHg in the presented case, well within the high-risk range.

### Pivotal role of blood pressure variability

Studies have demonstrated (Lucci et al., 2021) that autonomic nerve impairment can be evaluated using BPV. The most commonly used indicators for quantifying BPV are SD and CV of BP measurements, with CV providing additional information by partially correcting for the proportional association between mean BP and its variation (Chen et al., 2022).

This patient’s pre-ICH ABPM demonstrated extreme BPV (SBP SD 31.8 mmHg, CV 24.5%), exceeding proposed risk thresholds associated with poor ICH outcome (commonly SBP SD > 15 mmHg) (Manning et al., 2014; Jafari and Damani, 2020). High BPV may exacerbate vascular vulnerability by increasing cyclic shear stress on vessel walls, fostering inflammation, and inducing endothelial dysfunction (Manning et al., 2015; Jafari and Damani, 2020; Luo et al., 2024). The extreme BPV observed in this case illustrates the underlying pathophysiology of impaired baroreflex sensitivity and sympathetic dysregulation inherent to SCI (Rempel et al., 2025).

It is imperative to interpret the role of BPV in this case with caution. A similar credible interpretation is that the extreme BPV serves as a sensitive biomarker of the severity of underlying autonomic instability and baroreflex dysfunction associated with high-level SCI (Cowan et al., 2020).

### Emerging therapies and foundational prevention

Expanding on the standardized acute management discussed above, it is crucial to recognize that AD remains challenging to control in certain patients, necessitating investigation into novel therapeutic avenues. However, the following emerging therapies are in their early stage and do not replace foundational prevention strategies. AD is susceptible to recurrence and difficult to control when treated conventionally. Consequently, new strategies must be pursued. Cloutier employed olfactory ensheathing cell transplantation to address SCI and discovered that it did not mitigate the severity of AD attacks; however, it expedited BP recovery in approximately 50% of cases (Cloutier et al., 2016). This is initial animal research. Future research should focus on the therapeutic benefits of olfactory ensheathing cell transplantation for patients with chronic spinal cord injury, especially those with high thoracic spinal cord injury coupled with AD. Micaela LO’Reilly (O’Reilly et al., 2025) demonstrated that intrathecal administration of nuclear factor κB (NF-κB) inhibitors can mitigate AD, enhance survival, and offer a potential treatment strategy for cardiovascular and immune complications following high-level SCI. The Sachdeva R investigated a patient with cervical SCI and complete motor function loss; his autonomic nerve dysreflexia was alleviated after long-term application of transcutaneous spinal cord stimulation. Given the therapeutic potential of neuromodulation after SCI, this non-invasive pilot trial aims to address a critical gap in the literature and inform future applications of this stimulation approach after SCI (Sachdeva et al., 2021). Spinal cord electrical neuromodulation signifies a promising approach for enhancing autonomic control following SCI (Gadot et al., 2022; Chalif et al., 2024). Recently, Soriano presented a mechanistic framework by demonstrating that daily epidural electrical stimulation aimed at the lower thoracic spinal cord competitively reorganizes maladaptive autonomic circuitry, thereby reversing autonomic dysreflexia in animal models and mitigating its severity in humans with chronic spinal cord injury (Soriano et al., 2025). Current investigations on AD have yielded promising findings in neuromodulation, immunomodulation, and cell transplantation. However, prevention remains the fundamental principle. Enhanced bowel and bowel management, skin protection, and early treatment of infections can minimize the causes of AD.

### Aligning with current clinical practice for AD management

Notably, the current, evidence-based management of acute AD, as outlined by the Consortium for Spinal Cord Medicine (Krassioukov et al., 2021), remains the foundation of care. This entails prompt patient positioning (upright), identification and elimination of noxious stimuli, and continuous BP monitoring. For persistent severe hypertension, the use of rapid-onset, short-acting antihypertensive agents is recommended to reduce subsequent hypotension risk. In this case, although nitrendipine was administered and tolerated, guideline-preferred agents, including sublingual nifedipine or nitroglycerin spray, are generally favored in acute settings.

## Conclusion

Any acute severe headache in a patient with SCI at or above T6 necessitates immediate BP measurement and evaluation for ICH. Extreme BPV (SBP SD > 15 mmHg on 24 h ABPM) may indicate a subset of patients with severe autonomic instability necessitating increased monitoring and aggressive trigger avoidance. Structured bowel and bladder programs are essential for prevention. Future research should examine whether interventions designed to stabilize long-term BPV decrease cerebrovascular events in this high-risk population.

### Patient perspective

The patient provided written informed consent for the publication of this case report and shared his perspective on the experience:

“The headache was a sudden explosion in my head. I did not know that straining during bowel movement could precipitate a stroke. The doctors explained that my blood pressure was fluctuating markedly, not merely elevating. This clarification helped me understand why I sometimes felt lightheaded and at other times experienced severe headaches. Although the bowel management program was initially uncomfortable, it provided reassurance. I only wish someone had informed me about this risk months earlier, soon after my injury.”

### Strengths and limitations of this case report

This case report has several notable strengths. First, to our knowledge, this is the first study to provide quantitative pre-ICH BPV indices in a case of AD-related ICH. The availability of 24-h ambulatory blood pressure monitoring data both before the first event and during the interhemorrhagic interval represents a unique feature, enabling hypothesis-generating observation regarding BPV. Second, the case highlights a critical clinical insight: despite adherence to standard AD management and acute BP reduction, the patient experienced a recurrent ICH. These findings suggest that solely targeting the absolute reduction of episodic hypertensive peaks may be insufficient to prevent cerebrovascular recurrence. Notably, persistently elevated BPV was observed in this patient before the first ICH and in the interval between events, with systolic BP standard deviation (SD) consistently exceeding 30 mmHg. These findings support the hypothesis that extreme BPV may serve as a more sensitive marker of underlying autonomic instability and a potentially critical, under-recognized, therapeutic target in this population. Future studies should prioritize longitudinal BPV monitoring during AD management and explore novel interventions aimed at stabilizing BPV, including emerging neuromodulation techniques. Third, the case incorporates a comprehensive autonomic function assessment using the ISAFSCI and documents recurrent, anatomically distinct ICHs.

This study has several limitations. First, CAA could not be definitively excluded. The patient’s age (71 years) and lobar hemorrhage distributions are suggestive of CAA. However, MRI with SWI was unfeasible due to intolerance of prolonged supine positioning required for the examination due to severe orthostatic hypotension and pain, which precluded a motion-free study. Accordingly, CAA remains a possible contributing factor. Second, the causal role of BPV remains uncertain; it may reflect a biomarker of severe autonomic dysregulation inherent to high-level SCI, rather than an independent modifiable risk factor. Third, although two ABPM recordings were obtained (pre-event and inter-event), continuous, longitudinal BPV data over an extended period were lacking, and it remains unknown whether BPV fluctuates further in response to clinical interventions or time. Fourth, the generalizability of these findings is limited, as this report describes one elderly male with complete cervical SCI; applicability to younger patients or those with incomplete injuries remains uncertain. Fifth, potential confounders, including the patient’s pre-existing chronic hypertension and age-related vascular fragility, likely contributed to the overall ICH risk. The relative contribution of each factor cannot be delineated from this single case.

## Data Availability

The datasets presented in this study can be found in online repositories. The names of the repository/repositories and accession number(s) can be found in the article/supplementary material.
